# Objective assessment of physical activity and sedentary behaviour in knee osteoarthritis patients – beyond daily steps and total sedentary time

**DOI:** 10.1186/s12891-018-1980-3

**Published:** 2018-02-23

**Authors:** Maik Sliepen, Elsa Mauricio, Matthijs Lipperts, Bernd Grimm, Dieter Rosenbaum

**Affiliations:** 1 Institut für Experimentelle Muskuloskelettale Medizin (IEMM), Universitätsklinikum Münster (UKM), Westfälische Wilhelms-Universität Münster (WWU), Albert-Schweitzer Campus 1, Gebäude D3, 48129 Münster, Germany; 2 AHORSE, Department of Orthopaedics, Zuyderland Medical Centre, Henri Dunantstraat 5, 6419 PC Heerlen, the Netherlands; 3 Otto Bock Healthcare GmbH, Hermann-Rein-Str. 2a, 37075 Göttingen, Germany

**Keywords:** Knee osteoarthritis, Physical activity, Sedentary behaviour, Accelerometer, Body-worn sensors, F.I.T.T., Stair climbing, Objective assessment

## Abstract

**Background:**

Knee osteoarthritis patients may become physically inactive due to pain and functional limitations. Whether physical activity exerts a protective or harmful effect depends on the frequency, intensity, time and type (F.I.T.T.). The F.I.T.T. dimensions should therefore be assessed during daily life, which so far has hardly been feasible. Furthermore, physical activity should be assessed within subgroups of patients, as they might experience different activity limitations. Therefore, this study aimed to objectively describe physical activity, by assessing the F.I.T.T. dimensions, and sedentary behaviour of knee osteoarthritis patients during daily life. An additional goal was to determine whether activity events, based on different types and durations of physical activity, were able to discriminate between subgroups of KOA patients based on risk factors.

**Methods:**

Clinically diagnosed knee osteoarthritis patients (according to American College of Rheumatology criteria) were monitored for 1 week with a tri-axial accelerometer. Furthermore, they performed three functional tests and completed the Knee Osteoarthritis Outcome Score. Physical activity levels were described for knee osteoarthritis patients and compared between subgroups.

**Results:**

Sixty-one patients performed 7303 mean level steps, 319 ascending and 312 descending steps and 601 bicycle crank revolutions per day. Most waking hours were spent sedentary (61%), with 4.6 bouts of long duration (> 30 min). Specific events, particularly ascending and descending stairs/slopes, brief walking and sedentary bouts and prolonged walking bouts, varied between subgroups.

**Conclusions:**

From this sample of KOA patients, the most common form of activity was level walking, although cycling and stair climbing activities occurred frequently, highlighting the relevance of distinguishing between these types of PA. The total active time encompassed a small portion of their waking hours, as they spent most of their time sedentary, which was exacerbated by frequently occurring prolonged bouts.

In this study, event-based parameters, such as stair climbing or short bouts of walking or sedentary time, were found more capable of discriminating between subgroups of KOA patients compared to overall levels of PA and sedentary time. Thereby, subtle limitations in physical behaviour of KOA-subgroups were revealed, which might ultimately be targeted in rehabilitation programs.

**Trial registration:**

German Clinical Trials Registry under ‘DRKS00008735’ at 02.12.2015.

## Background

Knee osteoarthritis (KOA) is a frequent cause of pain and disability, affecting over 250 million people worldwide [1, 2]. In part due to experienced pain and limitations, KOA patients generally spend most of their waking hours sedentary (i.e. sitting or lying as opposed to standing or in locomotion) and fail to adhere to physical activity recommendations [3, 4]. Such behaviour could ultimately lead to harmful co-morbidities such as cardiovascular diseases [5].

Physical activity (PA) is a multidimensional behaviour, which is suggested to consist of four dimensions (F.I.T.T.: frequency, intensity, time and type) according to the World Health Organization (WHO) [6]. Whether PA exerts a protective or harmful effect seems to depend primarily on the type, intensity and frequency of PA [7], which highlights the importance of assessing the four dimensions of PA during daily life. On one hand, PA could lead to the progression of KOA, as heavy, repetitive loading may detrimentally affect the articular cartilage of the knee [7]. On the contrary, PA has been shown to prevent functional impairment and maintain independence in KOA patients [7].

A multitude of methods exists to assess PA during daily life. Arguably, the most appropriate method is using accelerometer-based activity monitors, as these are able to implement an activity event-based approach and thus objectively capture the F.I.T.T. dimensions of PA [8]. Furthermore, these devices can objectively determine the time spent sedentary, which is known to detrimentally affect the general health and functional status of KOA patients [4].

So far, studies that have assessed the physical behaviour of KOA patients with activity monitors have generally not been able to describe the four individual dimensions of PA, particularly the type of activities that were performed by KOA patients. Therefore, potentially relevant knowledge regarding the effect of different types of PA on KOA might have been missed. Due to recent technological advancements, the objective assessment of stair/slope locomotion and bicycling, in addition to stepping behaviour, is now feasible [9]. Furthermore, the time patterns of activity and sedentary behaviour can now be determined objectively [9]. Stair locomotion might be a clinically relevant assessment outcome in KOA, as it is often reported as one of the most demanding or even painful activities and is essential for the independence of patients in daily life [10]. Similarly, objectively quantifying bicycling in a free-living environment is important, since the joint moments experienced during cycling are considered small compared to other activities [11]. Cycling is therefore perceived as less painful and commonly prescribed as rehabilitating exercise in KOA patients [12]. In addition, bicycling is a common mode of transportation and recreational activity in Europe [13]. Interrupting sedentary behaviour has been shown to improve physical function and general health of older adults [14, 15]. Consequently, assessing the distribution of sedentary bout durations and interruptions, in addition to overall levels of sedentary time, should be a valuable measurement outcome in a KOA population.

Physical behaviour of KOA patients should not only be assessed on a population-level, but also within subgroups, as they are suggested to engage in different types of PA and experience varying activity limitations [16, 17]. Thus, differentiating between subgroups, which can be characterized based on risk factors (e.g. gender, BMI and knee injury) [18], might reveal subgroup-specific PA limitations. For this purpose, an event-based approach (e.g. activity bouts of specific durations and types) has been suggested to be more discriminative compared to general outcome measures that describe overall levels of PA (e.g. total amount of daily steps) [19].

Therefore, this study aimed to comprehensively describe physical activity, by assessing F.I.T.T. dimensions, and sedentary behaviour of knee osteoarthritis patients during daily life. An additional goal was to determine whether activity events, based on different types and durations of PA, were able to discriminate between subgroups of KOA patients based on risk factors.

## Methods

### Study design

A cross-sectional, exploratory study was conducted to assess physical activity, sedentary behaviour and physical function in German knee osteoarthritis patients during daily life.

### Participants

Eligible adults were required to present with clinical KOA (according to the guidelines of the American College of Rheumatology) and to report pain on most (≥4) days of the week for more than 3 months [20]. Individuals were excluded if they: (1) suffered from rheumatoid arthritis; (2) had a knee replacement, or were scheduled for replacement surgery within 3 months at the beginning of study participation; (3) suffered from medical conditions which could interfere with the activity and test performance, such as neurologic or severe cardiovascular conditions, (4) used an ambulatory aid for more than 50% of the ambulatory time. All participants were recruited at local hospitals and general practices.

### Physical activity assessment

Physical activity was monitored using the AX3, a small tri-axial accelerometer (23 × 32.5 × 7.6 mm; sampling frequency: 50 Hz; Axivity Ltd., Newcastle, UK). Patients were instructed to wear the device for seven consecutive days during waking hours, except for water-based activities. The device was worn on the lateral side of the right thigh, halfway between femoral head and tibial plateau, attached by double-adhesive tape. To ensure consistency, the researcher marked the wear location.

Relevant parameters of the F.I.T.T. dimensions were extracted using custom-developed Matlab algorithms (detailed information has been described elsewhere [9]), which have shown highly accurate results in both healthy participants and orthopaedic lower-extremity patients [9]. In short, various types of activities were assessed (i.e. walking, sitting, standing, stair/slope climbing and bicycling). Furthermore, the frequency with which these activities were performed and the time spent within the activities was monitored. The intensity of walking (i.e. walking cadence) was also determined (Table 1). Participants filled in a daily log reporting on wear times, experienced pain through a numeric rating scale (NRS; 0 is no pain, 10 is worst conceivable pain) and physical activity events that could not be monitored with the algorithms (i.e. resistance exercise, swimming, gardening and household activities).

**Table 1:** Parameters extracted from AX3, categorized according to F.I.T.T. acronym

Dimension	Parameter
*Frequency*	Number of level steps Number of ascending steps Number of descending steps Number of bicycling crank revolutions Bouts of level steps Bouts of ascending steps Bouts of descending steps Number of walking bouts (0–5 s) Number of walking bouts (5–10 s) Number of walking bouts (300–600 s) Number of walking bouts (> 600 s) Number of sedentary bouts (0–10 s) Number of sedentary bouts (10–60 s) Number of sedentary bouts (1200–1800 s) Number of sedentary bouts (> 1800 s)
*Intensity*	Walking cadence
*Time*	Time spent walking (incl. Level, ascend and descend). Time spent bicycling Time spent sitting Time spent standing
*Type*	Walking Stair/slope ascending Stair/slope descending Bicycling Sitting Standing STS transfers

*STS* Sit-to-stand

### Physical function scores

The participants performed three functional tests, rather than a single one, in order to represent different activities of daily life. They performed the 40 m (4 × 10 m) fast-paced Walk Test (WT), Timed Up and Go Test (TUGT) and 15-Stair Climb Test (SCT). The tests are proven to be valid, reliable and sensitive for knee OA patients [21]. Tests were conducted following the standard protocol instructing patients to perform as fast as possible while the time was recorded with a stopwatch [21]. Each test was performed three times and the average time was used for further analysis.

### Questionnaire

Patients were asked to fill in the Knee Osteoarthritis Outcome Score (KOOS), a valid and responsive questionnaire covering several disease-related domains, ranging from 0 (most severely affected) to 100 (not affected) [22]. Furthermore, patients were asked about a previous knee injury (‘Have you ever had a knee injury, which resulted in the inability to walk for over one week’), their job and employment status (i.e. employed, unemployed or retired) and whether they performed any sports on a regular basis (specifics regarding type of sports and frequency).

### Statistical analysis

After the measurement, all participant data was pseudonymised. For the activity monitor data, a minimum of 4 valid wear days (i.e. at least 10 daily wear hours) was used as threshold, as these are minimally needed to obtain reliable PA estimates [23, 24]. Participants with insufficient valid wear days were excluded from further analysis.

All analyses were performed with SPSS (Version 23, SPSS Inc., USA), with the significance level set at α = 0.05. First, descriptive statistics regarding socio-demographics, health-related factors and activity parameters were calculated for the total patient sample. Spearman’s rank correlations were computed between activity parameters, the function test scores and KOOS outcomes.

Next, patients were grouped using their respective gender (male/female), BMI category (normal, BMI < 25; overweight, BMI 25–30; obese, BMI > 30) and history of knee injury (knee injury/no knee injury) as a variable. Data was tested for normality using the Shaphiro-Wilk Test and distribution histograms. In case of positively-skewed data (i.e. a violation of the normality-assumption), either a square-root or logarithmic transformation was used to generate a normal distribution [25]. One-way ANCOVA’s were then used to examine the difference in PA parameters between subgroups of KOA patients, while adjusting for possible confounders (i.e. age, gender, BMI, pain and knee injury) [26]. A post-hoc Bonferroni correction was incorporated to adjust for multiple comparisons [25]. Afterwards, the transformed data was back-transformed to present meaningful values [25]. If the data was extremely skewed and a normal distribution could not be achieved (e.g. data that frequently contains ‘0’), non-parametric tests were used (e.g. Mann-Whitney U Test). Data regarding the socio-demographics, health factors and physical function of the complete sample were presented as mean ± standard deviation (SD). The differences between subgroups were presented as adjusted mean ± standard error (SE). Finally, transformed data was presented as adjusted mean (95% confidence interval (CI)) [25].

## Results

### Socio-demographics, health factors and physical function

In total, 61 of the included 64 participants provided valid activity data and were included for further analysis (56% female). The average age was 60 (±10) years. Of the included patients, 18, 30 and 13 were categorized as having a ‘normal’, ‘overweight’ and ‘obese’ BMI, respectively. They reported a mean pain score of 3.0 (NRS) or 57.4 (KOOS_pain_). A slight majority of the patients (51%) had previously suffered a knee injury (Table 2). Most were still employed (57%) and 56% of the participants reported to perform some form of sports, twice per week. With respect to the functional tests, the patients needed a mean time of 26.1 (±4.7), 7.2 (±1.6) and 14.0 (±4.9) seconds to complete the WT, TUGT and SCT respectively (Table 2).

**Table 2:** Participant characteristics, physical function and knee osteoarthritis outcome scores (*n* = 61)

Characteristics	Mean (±SD)	Range
*Sociodemographics*		
Age (years)	60.7 (10.0)	37.0–79.0
Gender (male/female)	27/34	
Employment status (employed/retired)	35/26	
Regular sport participation (yes/no)	34/27	
*Health factors*		
BMI (kg/m^2^)	27.3 (4.7)	19.7–42.8
Injury history (yes/no)	31/30	
Pain (NRS)	3.0 (1.5)	1.0–7.8
*Physical function tests*		
40 m Walk Test (s)	26.1 (4.7)	19.1–37.1
Timed Up and Go Test (s)	7.2 (1.6)	4.5–12.0
Stair Climb Test (s)	14.0 (4.9)	7.6–37.2
*Knee Osteoarthritis Outcome Score*		
Pain	57.4 (20.5)	5.6–96.4
Symptoms	55.0 (20.3)	14.3–96.4
Activities of daily living	64.1 (21.3)	0–100
Sports	36.1 (25.3)	0–100
Quality of life	31.0 (19.1)	0–81.3

*BMI* Body Mass Index, *NRS* Numeric Rating Scale

### Physical activity and sedentary parameters

The AX3 was worn for a mean of 6.5 (±1.0) days, with an average wear time of 14.7 (±1.2) hours per day. Patients spent 11% of the waking hours walking (incl. Stair locomotion) and 1% bicycling, whereas most of the time was spent with non-locomotion behaviour (88%).

A mean amount of 7934 (±2326) steps were recorded per day, with an average cadence during walking bouts of 100 (±11) steps per minute. A quarter of the participants performed prolonged walking periods (lasting more than 10 min), twice per week. Ascending and descending stairs or slopes occurred 25 and 22 times on a daily basis, with a large variation ranging from 2 to 64 bouts (Table 3). The majority of participants (72%) cycled during the measurement period for a mean of 20 (±17) minutes per day. If crank revolutions were added as steps (since step-based PA recommendations do not discriminate between walking and cycling behaviour) 8535 steps would be counted on a daily basis. This way 25% of the participants would reach the commonly advocated threshold of 10,000 daily steps [3].

**Table 3:** Physical activity outcomes, extracted from the AX3 (*n* = 61)

Parameter	Mean (±SD)	Range
Total wear time	14. 7 (1.2)	11.7–17.6
*Time spent (h)*		
Sitting	8.9 (1.8)	4.7–13.3
Standing	4.0 (1.4)	1.3–9.2
Walking	1.6 (0.4)	0.4–2.4
Bicycling	0.2 (0.3)	0.0–1.3
*Bouts of steps (n)*		
Level	232 (66)	48–379
Up	25 (15)	4–64
Down	22 (14)	2–55
*Amount of steps (n)*		
Total	7934 (2326)	1902–13,560
Level	7303 (2137)	1611–12,984
Up	319 (288)	15–1255
Down	312 (343)	18–2280
*Cycling*		
Crank revolutions	601 (754)	0–3959
*Walking periods (n)*		
0–5 s	4.4 (2.7)	0.0–10.7
5–10 s	61.2 (22.4)	10.2–123.2
300–600 s	0.5 (0.5)	0.0–1.9
> 600 s	0.1 (0.2)	0.0–0.8
*Sedentary periods (n)*		
0–10 s	3.6 (2.7)	0.38–12.0
10–60 s	15.4 (9.1)	3.3–37.7
1200–1800 s	2.6 (0.9)	0.6–4.6
> 1800 s	4.6 (1.7)	1.2–9.2
STS transfers (*n*)	52 (18)	26–105
Cadence (steps/min)	100.3 (10.6)	74.0–136.8

*STS* Sit-to-stand

Non-locomotion time (mean: 12.9 h/day) primarily comprised of sedentary behaviour (69%) in comparison with standing (31%). On average, 52 sit-to-stand (STS) transfers were performed per day. Continuous sedentary periods lasting between 20 and 30 min and longer than 30 min occurred 2.6 (±0.9) and 4.6 (±1.7) times per day, resp. (Table 3).

The time spent within activities or sedentary behaviour was not associated with functional test outcomes (*ρ* < 0.21, *p* > 0.11). Furthermore, associations between the total amount of daily steps and functional test outcomes were insignificant or weak at best (*ρ* < 0.40, *p* ≥ 0.05). However, the amount of ascending/descending steps (*ρ* = − 0.55 to − 0.68), short activity periods (max. 5 s, *ρ* = − 0.61 to − 0.64) and short sedentary periods (max. 10 s, *ρ* = − 0.58 to − 0.62) were significantly associated with the time taken to complete the functional tests (*p* < 0.001).

### Comparison of subgroups based on gender

No significant difference was found in the amount of daily steps between male and female participants (*p* = 0.89). However, the male participants performed 81% more ascending steps (adjusted mean (CI): 294 (250, 344) vs. 162 (141, 186), *p* < 0.01) and 84% more descending steps (adjusted mean (CI): 269 (229, 316) vs. 146 (127, 168), *p* < 0.01) during daily life. In addition, male participants completed the SCT significantly faster compared to females (adjusted mean ± SE: 12.2 ± 0.8 vs. 15.5 ± 0.7, *p* < 0.01). Males spent a larger proportion of waking hours sedentary (adjusted mean ± SE: 64 ± 2% vs. 57 ± 2%, *p* = 0.02) compared to females. This difference was highlighted by more prolonged sedentary bouts lasting longer than 30 min (adjusted mean ± SE: 5.2 ± 0.3 vs. 4.2 ± 0.3 per day, *p* = 0.03). Females spent more time standing (adjusted mean ± SE: 30 ± 2% vs. 23 ± 2%, *p* < 0.01).

### Comparison of subgroups based on BMI

**Fig. 1:**
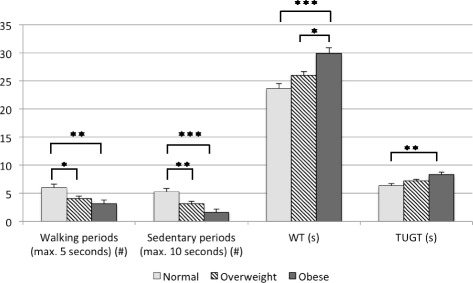
The difference in activity parameters between healthy, overweight and obese KOA patients. Note: * = *p* < 0.05, ** = *p* < 0.01, *** = *p* < 0.001. Abbreviations: 40 m fast-paced Walk Test (WT) and Timed-Up-and-Go Test (TUGT)

Between the BMI categories, no significant differences were found in the time spent walking, cycling, standing or sitting (*p* > 0.55). Normal-weight KOA patients walked more steps on a daily basis than overweight KOA patients (adjusted mean ± SE: 8974 ± 558 vs. 7153 ± 420 steps/day, *p* = 0.04). However, no significant differences in daily steps were found between the other subgroups (normal vs. obese and overweight vs. obese, *p* > 0.43). Normal-weight participants performed 158% more ascending steps (adjusted mean (CI): 341 (281, 413) vs. 132 (106, 164), *p* < 0.01) and 175% more descending steps (adjusted mean (CI): 375 (326, 429) vs. 117 (152, 86), *p* < 0.001) compared to obese individuals. Furthermore, significant differences were found between the amount of brief walking bouts (up to 5 s) and short sedentary periods (max. 10 s) (Fig. 1). These differences were present between the normal vs. overweight and normal vs. obese group. During the functional tests, the obese participants needed more time to finish the WT and TUGT compared to the normal-weight participants. They also performed the WT significantly slower than the overweight individuals (Fig. 1).

### Comparison of subgroups based on previously experienced knee injury

The majority of the activity and functional parameters was comparable between the participants with and without a knee injury history. Yet, the patients, who had previously suffered from a knee injury, performed fewer continuous walking bouts lasting more than 10 min (median (interquartile range): 0.00 (0.14) vs. 0.14 (0.34), *p* < 0.01), compared to the group with no injury history. They also tended to report lower scores on the KOOS-subscale related to symptoms (adjusted mean ± SE: 49.9 ± 3.3 vs. 60.7 ± 3.4, *p* = 0.03).

## Discussion

Although PA and sedentary behaviour of KOA patients have been examined before, previous studies have not objectively investigated the four dimensions of PA, particularly the type of activities performed during daily life. The included KOA patients accumulated 7303 mean level steps, representing the majority of daily PA. In addition, most of the patients engaged in bicycling for which time and crank revolutions were measured. Mean steps for ascending and descending stairs or slopes, which comprised a significant part of the overall step count, were also assessed. The daily PA was executed during a small portion of the waking hours, since these were primarily spent sedentary, mainly through prolonged bouts (> 30 min). Furthermore, subgroups of KOA patients, based on risk factors (i.e. gender, BMI and history of knee injury), showed only few significant differences in general activity parameters. However, event-based parameters, such as the amount of ascending and descending steps and short bouts of walking or sedentary time (< 10 s), were significantly different in most of the subgroup-comparisons.

Overall, KOA patients walked 7934 steps (including level, ascending and descending steps) on a daily basis. These findings compare well with the results of a recent systematic review, including over 3000 KOA patients that averaged 7750 daily steps [3]. The review included patients who varied extensively with respect to the severity of disease (including early-stage KOA up to pre-TKA patients) and country of origin (including countries from Europe, USA and Asia). Most of the participants (75%) did not perform prolonged walking activities (lasting over 10 min), which are suggested to be health-relevant [3]. The individuals that engaged in such continuous walking activity averaged only 2 bouts per week, whereas 3 daily bouts are recommended in PA guidelines [3]. KOA patients ascended and descended stairs or slopes regularly (25 and 22 times per day, resp.), even though it is regarded as one of the most demanding and potentially painful activities of daily life [10]. The large inter-participant variation might indicate that some individuals only engage in stair locomotion if necessary (e.g. within one’s home), whereas others engage in it voluntarily and consciously, possibly for exercise purposes (e.g. during commuting or at work). In the latter example, stair climbing might have been avoided by taking the elevator, although this option is not always available. Furthermore, the majority of participants (72%) cycled during the measurement period for 20 min per day, which probably occurred as bicycling is a common mode of transportation and recreational activity in Europe [13]. Although level walking is the most common form of activity (85%), cycling (7%), ascending (4%) and descending stairs and slopes (4%) in addition occurred frequently in this sample of KOA patients, which underlines the importance of distinguishing between various types of PA.

Sedentary behaviour was commonly observed in the majority of the patients. To emphasize, 60% of the waking time was spent sedentary, which is slightly below the percentage reported by other studies (65–66%) [5, 27]. The sedentary time is accumulated to a large degree during long bouts (longer than 30 min), which are suggested to detrimentally affect general health [15]. The continuous bouts occurred more often in this population (4.6 times per day) compared to previous reports of pre- and post-total knee arthroplasty (TKA) patients (3.4 and 3.1, respectively) [5]. The variations in total sedentary time and amount of prolonged sitting bouts, however, might have occurred due to differences in applied activity monitors and methods [28]. When examining the sit-to-stand transfers, KOA patients performed 52 transitions per day, which compares well with the results of a recent review, reporting a range of 45 to 71 STS transfers [29]. Within the review, a large variety of participant groups were included, varying from healthy older adults to cancer patients. This suggests that KOA patients are still equally able to perform STS-transfers compared to other populations, which is important for maintaining independence during daily life [29].

This study also aimed to determine whether activity events, based on different types and durations of PA, were able to discriminate between subgroups of KOA patients. Such subgroups might engage in different types of PA during daily life and experience varying activity limitations [16, 17], which would be missed if the population were only analysed as a whole.

No gender differences were found in the number of daily steps. However, the male participants engaged in stair climbing more regularly during daily life compared to females. Males are known to ascend and descend stairs at higher speeds and are thus suggested to possess a superior physical capacity to climb stairs [30]. Stair climbing is known to be a challenging activity, especially for individuals with a poorly functioning lower-extremity [31]. Females, who generally have a poorer physical function than males, might therefore have tried to avoid stair climbing during daily life (e.g. by taking an elevator). Furthermore, the male individuals spent more waking hours sedentary, by engaging in prolonged sedentary bouts (> 30 min) more frequently, and less hours standing compared to females. This difference did not occur due to employment status, as the amount of employed males and females was comparable (*p* = 0.80). In addition to their occupation, females however engage in domestic activities more regularly [32]. Such activities (e.g. ironing and cooking) do not necessarily affect the amount of PA, but will reduce sedentary time. These findings show that male individuals climb more stairs and slopes and perform better on functional tests, but are also more sedentary compared to women, which confirms that PA and sedentary behaviour are two distinct dimensions that should be assessed separately [33].

Normal-weight patients walked more steps during daily life than overweight patients, which seems to confirm that BMI is negatively related to daily steps [34]. Unexpectedly, no differences in daily steps were found between the other subgroups. A possible explanation might be that the levels of PA were affected through factors that were not incorporated within this study. Psychological barriers, such as embarrassment, lack of motivation and the fear of experiencing pain upon being physically active, have been suggested to greatly affect the physical behaviour of KOA patients [35, 36].

Significant differences were also found in the amount of ascending and descending steps between the ‘normal-weight’ and ‘obese’ subgroup. During stair climbing, knee loading is more demanding in obese individuals [37]. As a result, they appear to minimize this kind of loading by engaging in fewer ascending and descending steps. Obese and overweight patients furthermore performed significantly less brief sedentary periods than normal-weight participants. STS transfers are suggested to be more challenging for these subgroups [38]. Consequently, they might attempt to avoid short sitting bouts (up to 10 s) and remain standing, thereby avoiding the need for sit-to-stand loading in a brief time period. Unexpectedly, the 27% difference in STS transfers was found to be insignificant, which could be due to the relative small subsamples (18 normal-weight, 30 overweight and 13 obese patients). In addition, normal-weight participants performed more brief walking bouts (up to 5 s) than the overweight and obese individuals. Perhaps, they should not be described as short-lasting walking bouts, yet more as an individual activity category. Household activities, such as cooking, gardening or cleaning, would probably include many of these short activity bouts. Normal-weight patients might be more likely to perform such activities, thereby explaining that a high amount of these walking bouts (which hardly affect the total step count) occurred more frequently in this subgroup. Examining activity events, based on different types and durations of PA seems to reveal differences between BMI categories that could not be revealed whilst assessing the total amount of daily steps or sedentary time.

A knee injury is strongly related to the progression of KOA and increases the risk of developing disease-related pain and symptoms [39]. In the current population, this seems to have resulted in a reduced amount of long-lasting activities (min. 10 min) in patients that have experienced a knee injury. Unexpectedly, both groups reported comparable pain levels. Pain scores, such as the NRS, might be inadequate to properly capture the complexity and fluctuations of pain in KOA, as they assess only average levels of pain and do not specify the experienced pain during such a particular activity (i.e. prolonged walking of at least 10 min) [40]. Patients with a history of knee injury also reported to suffer from more severe disease-related symptoms, as has been suggested previously [39]. The difference between the two groups was considered clinically relevant, since they exceed the difference of 8 to 10 points on the KOOS-scale [41].

In general, the total amount of daily steps or time spent within activities and postures (e.g. sedentary time) did not differ between subgroups of KOA patients. In addition, daily steps and time spent within activities were at best weakly associated with physical function. However, event-based parameters (e.g. stair climbing and brief periods of activity or sedentary behaviour) were shown to discriminate between subgroups of KOA patients more adequately. The significant association between the same parameters and physical function strengthened these findings. Thus, event-based parameters have an enhanced discriminatory capacity, not only between subgroups, but also with respect to physical function, compared to parameters of overall PA and sedentary behaviour. Furthermore, subtle limitations in physical behaviour of KOA-subgroups were revealed. To our knowledge, this has not been reported previously in this population. Although these findings need to be confirmed in future studies, these activity limitations might ultimately be targeted in rehabilitation programs to aid in maintaining the independence of KOA patients.

Several limitations should be acknowledged. First, radiographic imaging was not available for all participants, which resulted in the inability to grade the structural degeneration of the joint using common radiographic scales. Therefore, this study was unable to assess the physical behaviour of different grades of KOA. Although differences could be expected, a previous review reported comparable PA levels between patients with mild and severe KOA [3]. This however, might have occurred as only overall levels of PA were assessed and the included studies did not report specific types or durations of activities. Secondly, due to the design of the study, the same outcome measures have been compared and analysed multiple times, thereby possibly inducing family-wise errors [42]. Therefore, a Bonferroni correction was implemented in the ANCOVA’s [25]. It should be noted that no consensus currently exists whether *p*-value adjustments are preferential, mainly because a reduction in the chance of type-I errors will lead to an increased probability of type-II errors [42, 43]. Thirdly, we were able to control for confounding parameters in the majority of analysed activity parameters. However, two activity parameters (walking bouts lasting between 5 to 10 min and walking bouts lasting more than 10 min) generally did not occur amongst the majority of the patients. Therefore, their distribution was extremely positively-skewed and could not be transformed to achieve a normal distribution. As a result, ANCOVA’s could not be performed with these two parameters, so that we were not able to control for confounding variables.

This study included a relatively small patient sample, which might have reduced the odds of detecting a true effect [44]. For example, obese patients were found to perform less brief sedentary periods compared to normal-weight individuals. Surprisingly, there was no significant difference between the amounts of STS transfers performed by these subgroups (*p* > 0.25), although the difference between group means was 27%. This lack of significance might have occurred due to the small subgroup samples. It should be noted that the included participants were similar with respect to BMI, age and gender ratio compared to the average values of a recent systematic review, including 3266 KOA patients from 21 different studies [3]. Therefore this sample was considered comparable to other KOA studies. Nonetheless, this study’s findings cannot be generalized towards every other KOA population. For example, non-European KOA patients might cycle significantly less, as bicycling is less common in other continents [13].

Due to the wording of the injury question, mild knee injuries (e.g. small meniscal tears) might have been missed. Yet, more severe knee injuries, which are known to increase the risk of developing KOA [45], have most probably been captured. Finally, some limitations that can occur while examining PA with accelerometers should be recognized. Not all types of physical activity (i.e. water-based activities and strengthening exercises) can correctly be captured [46]. In addition, patients might adjust their habitual behaviour as a response to wearing an accelerometer (e.g. due to social desirability), although this effect is suggested to last only briefly in general [46]. Nonetheless, accelerometry is considered an objective and accurate method for monitoring PA during daily life [46].

Future studies with larger samples are needed to confirm the specific PA limitations that seemed to be present in this population. In addition, radiographic data should be included to comprehensively assess the differences in physical behaviour between patients with different KOA severities.

## Conclusions

In this sample of German KOA patients, the most common form of activity was level walking, although cycling and stair climbing activities occurred frequently, highlighting the relevance of distinguishing between these types of PA. The total active time encompassed only a small portion of their waking hours, as they spent most of their time sedentary, which was exacerbated by frequently occurring prolonged sedentary bouts.

In this study, event-based parameters, such as stair climbing or short bouts of walking or sedentary time, were found more capable of discriminating between subgroups of KOA patients compared to overall levels of PA and sedentary time. Thereby, subtle limitations in physical behaviour of KOA-subgroups were revealed, which might ultimately be targeted in rehabilitation programs to aid in maintaining the independence of KOA patients.

## Abbreviations

BMI: Body-mass index
CI: Confidence Interval
F.I.T.T: Frequency, intensity, time and type
KOA: Knee osteoarthritis
KOOS: Knee osteoarthritis outcome score
NRS: Numeric rating scale
PA: Physical activity (PA)
SCT: 15-Stair Climb Test
SD: Standard deviation
SE: Standard error
STS: Sit-to-stand
TKA: Total Knee Arthroplasty
TUGT: Timed up and Go Test
WHO: World Health Organization
WT: 40 m (4 × 10 m) fast-paced Walk Test
